# Bis[(1-vinyl-1*H*-imidazol-2-yl-κ*N* ^3^)methanamine-κ*N*]copper(II) bis­(hexa­fluoridophosphate)

**DOI:** 10.1107/S1600536811050100

**Published:** 2011-11-25

**Authors:** Alexander Schiller, Rosario Scopelliti, Wolfgang Imhof

**Affiliations:** a Institute for Inorganic and Analytical Chemistry, Friedrich Schiller University, Humboldtstrasse 8, 07743 Jena, Germany; b Institut des Sciences et Ingénierie Chimiques, Ecole Polytechnique Fédérale de Lausanne (EPFL), BCH-LCS, CH-1015 Lausanne, Switzerland

## Abstract

In the title compound, [Cu(C_6_H_9_N_3_)_2_](PF_6_)_2_, the Cu atom is located on a crystallographic center of inversion. The coordination environment of the Cu atom is square-planar with two amino and two imidazole N atoms bonded to the metal in a *trans* configuration.

## Related literature

For the title ligand as a building block for tripodal tetra­amine ligands, see: Blackman (2005 ▶). For catalytic activity of copper(II) complexes with similar mulidendate *N*-donor ligands, see: Schiller *et al.* (2005 ▶, 2006 ▶).
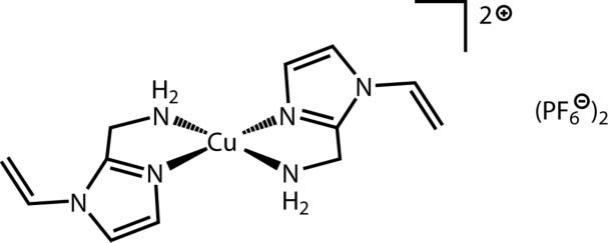


## Experimental

### 

#### Crystal data

[Cu(C_6_H_9_N_3_)_2_](PF_6_)_2_
*M* *_r_* = 599.80
Monoclinic, 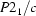

*a* = 11.543 (2) Å
*b* = 12.282 (2) Å
*c* = 8.2793 (14) Å
β = 96.476 (15)°
*V* = 1166.3 (4) Å^3^
*Z* = 2
Mo *K*α radiation
μ = 1.18 mm^−1^
*T* = 140 K
0.24 × 0.20 × 0.16 mm

#### Data collection

Oxford Diffraction KM-4/Sapphire CCD diffractometer
Absorption correction: multi-scan (Blessing, 1995 ▶) *T*_min_ = 0.657, *T*_max_ = 1.000
6439 measured reflections
1955 independent reflections
1396 reflections with *I* > 2σ(*I*)
*R*_int_ = 0.088

#### Refinement

*R*[*F* ^2^ > 2σ(*F* ^2^)] = 0.047
*wR*(*F* ^2^) = 0.123
*S* = 0.97
1955 reflections
151 parameters
H-atom parameters constrained
Δρ_max_ = 0.91 e Å^−3^
Δρ_min_ = −0.46 e Å^−3^

### 

Data collection: *CrysAlis CCD* (Oxford Diffraction, 2006 ▶); cell refinement: *CrysAlis RED* (Oxford Diffraction, 2006 ▶); data reduction: *CrysAlis RED*; program(s) used to solve structure: *SHELXS97* (Sheldrick, 2008 ▶); program(s) used to refine structure: *SHELXL97* (Sheldrick, 2008 ▶); molecular graphics: *SHELXTL* (Sheldrick, 2008 ▶); software used to prepare material for publication: *ORTEP-3* (Farrugia, 1997 ▶).

## Supplementary Material

Crystal structure: contains datablock(s) I, global. DOI: 10.1107/S1600536811050100/fi2116sup1.cif

Structure factors: contains datablock(s) I. DOI: 10.1107/S1600536811050100/fi2116Isup2.hkl

Additional supplementary materials:  crystallographic information; 3D view; checkCIF report

## Figures and Tables

**Fig. 1.**
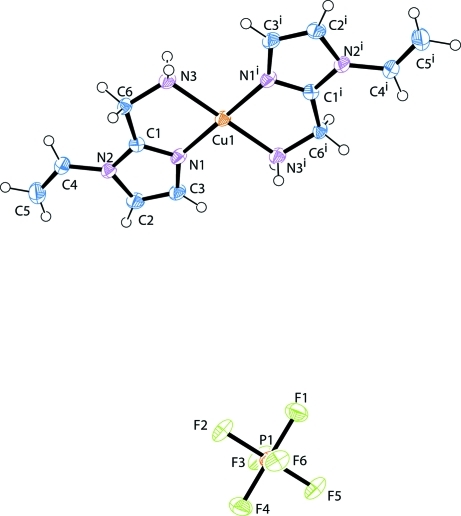
Molecular structure of the title compound. Second ligand is created by (i): -x, -y, -z. Ellipsoids are depicted on the 50% probability level.

## supplementary crystallographic information

### Comment

The described ligand has been used as a building block for the synthesis of the chelate ligand tris[(1-vinylimidazole-2-yl)methyl]amine (*L*). The complexes [Zn(*L*)Cl]PF_6_ and [Cu(*L*)Cl]PF_6_ were obtained upon reaction with *L* and immobilized by co-polymerization with ethylene glycol dimethacrylate. The supported complexes were found to be efficient heterogenous catalysts for the hydrolysis of bis(*p*-nitrophenyl)phosphate (Schiller *et al.*, 2006).

The structure of the title compound feature Cu on an inversion centre (Wyckoff position 2a). Two ligands coordinate to it in a trans fashion (Fig. 1).

### Experimental

Synthesis of the metal complex. Anhydrous copper(II) chloride (25.0 mg, 0.186 mmol) was added to a solution of (1-vinyl-1*H*-imidazol-2-yl)-methylamine (45.8 mg, 0.372 mmol) in ethanol (4 ml). NH_4_PF_6_ (60.6 mg, 0.372 mmol) was added and pink crystals were formed after 2 h. The product was isolated, washed with ethanol, and dried in a vacuum (yield: 83.7 mg, 75%). IR: ν (cm^-1^) = 3368/3314/3205 (w, NH), 1652 (*vs*, CH=CH_2_), 822 (*vs*, PF_6_).

### Refinement

Hydrogen atoms have been placed in calculated positions with C–H distances of 0.99Å for the methylene group and 0.95Å for all other hydrogen atoms bonded to carbon and 0.92Å for the amino function. Refinement was performed using a riding model with *U*_iso_(H) = 1.2 *U*_eq_(C).

### Crystal data

**Table:**

[Cu(C_6_H_9_N_3_)_2_](PF_6_)_2_	*F*(000) = 598
*M**_r_* = 599.80	*D*_x_ = 1.708 Mg m^−^^3^
Monoclinic, *P*2_1_/*c*	Mo *K*α radiation, λ = 0.71073 Å
Hall symbol: -P 2ybc	Cell parameters from 3520 reflections
*a* = 11.543 (2) Å	θ = 2.4–26.6°
*b* = 12.282 (2) Å	µ = 1.18 mm^−^^1^
*c* = 8.2793 (14) Å	*T* = 140 K
β = 96.476 (15)°	Prismatic, pink
*V* = 1166.3 (4) Å^3^	0.24 × 0.20 × 0.16 mm
*Z* = 2	

### Data collection

**Table:**

Oxford Diffraction KM-4/Sapphire CCD diffractometer	1955 independent reflections
Radiation source: fine-focus sealed tube	1396 reflections with *I* > 2σ(*I*)
graphite	*R*_int_ = 0.088
φ and ω scans	θ_max_ = 25.0°, θ_min_ = 3.0°
Absorption correction: multi-scan (Blessing, 1995)	*h* = −13→13
*T*_min_ = 0.657, *T*_max_ = 1.000	*k* = −14→14
6439 measured reflections	*l* = −9→9

### Refinement

**Table:**

Refinement on *F*^2^	Primary atom site location: structure-invariant direct methods
Least-squares matrix: full	Secondary atom site location: difference Fourier map
*R*[*F*^2^ > 2σ(*F*^2^)] = 0.047	Hydrogen site location: inferred from neighbouring sites
*wR*(*F*^2^) = 0.123	H-atom parameters constrained
*S* = 0.97	*w* = 1/[σ^2^(*F*_o_^2^) + (0.0677*P*)^2^] where *P* = (*F*_o_^2^ + 2*F*_c_^2^)/3
1955 reflections	(Δ/σ)_max_ < 0.001
151 parameters	Δρ_max_ = 0.91 e Å^−^^3^
0 restraints	Δρ_min_ = −0.46 e Å^−^^3^

### Special details

**Table:**

Geometry. All e.s.d.'s (except the e.s.d. in the dihedral angle between two l.s. planes) are estimated using the full covariance matrix. The cell e.s.d.'s are taken into account individually in the estimation of e.s.d.'s in distances, angles and torsion angles; correlations between e.s.d.'s in cell parameters are only used when they are defined by crystal symmetry. An approximate (isotropic) treatment of cell e.s.d.'s is used for estimating e.s.d.'s involving l.s. planes.
Refinement. Refinement of *F*^2^ against ALL reflections. The weighted *R*-factor *wR* and goodness of fit *S* are based on *F*^2^, conventional *R*-factors *R* are based on *F*, with *F* set to zero for negative *F*^2^. The threshold expression of *F*^2^ > σ(*F*^2^) is used only for calculating *R*-factors(gt) *etc*. and is not relevant to the choice of reflections for refinement. *R*-factors based on *F*^2^ are statistically about twice as large as those based on *F*, and *R*- factors based on ALL data will be even larger.

### Fractional atomic coordinates and isotropic or equivalent isotropic displacement parameters (Å^2^)

**Table:**

	*x*	*y*	*z*	*U*_iso_*/*U*_eq_	
Cu1	0.0000	0.0000	0.0000	0.0228 (2)	
N1	0.1254 (3)	0.1059 (2)	−0.0384 (4)	0.0259 (8)	
N2	0.3038 (3)	0.1375 (2)	−0.1192 (4)	0.0274 (8)	
N3	0.1108 (3)	−0.1091 (2)	−0.0966 (4)	0.0266 (8)	
H3A	0.1138	−0.1725	−0.0370	0.032*	
H3B	0.0811	−0.1258	−0.2015	0.032*	
C1	0.2219 (3)	0.0595 (3)	−0.0873 (5)	0.0222 (9)	
C2	0.2538 (4)	0.2409 (3)	−0.0848 (6)	0.0315 (11)	
H2	0.2886	0.3104	−0.0940	0.038*	
C3	0.1460 (4)	0.2200 (3)	−0.0360 (5)	0.0296 (10)	
H3	0.0933	0.2736	−0.0054	0.036*	
C4	0.4149 (4)	0.1189 (3)	−0.1822 (5)	0.0316 (10)	
H4	0.4311	0.0471	−0.2160	0.038*	
C5	0.4952 (4)	0.1947 (4)	−0.1960 (7)	0.0474 (14)	
H5A	0.4821	0.2675	−0.1634	0.057*	
H5B	0.5658	0.1763	−0.2383	0.057*	
C6	0.2332 (3)	−0.0645 (3)	−0.0967 (5)	0.0227 (9)	
H6A	0.2661	−0.0860	−0.1974	0.027*	
H6B	0.2847	−0.0924	−0.0021	0.027*	
P1	0.19988 (10)	0.57233 (7)	0.90769 (13)	0.0244 (3)	
F1	0.0883 (2)	0.51137 (19)	0.8082 (3)	0.0463 (8)	
F2	0.2875 (2)	0.50369 (17)	0.8029 (3)	0.0392 (7)	
F3	0.1923 (3)	0.66853 (17)	0.7698 (3)	0.0472 (8)	
F4	0.3113 (2)	0.6338 (2)	1.0077 (3)	0.0531 (8)	
F5	0.1112 (2)	0.64221 (17)	1.0131 (3)	0.0396 (7)	
F6	0.2065 (2)	0.47717 (17)	1.0447 (3)	0.0365 (7)	

### Atomic displacement parameters (Å^2^)

**Table:**

	*U*^11^	*U*^22^	*U*^33^	*U*^12^	*U*^13^	*U*^23^
Cu1	0.0264 (4)	0.0165 (3)	0.0261 (5)	0.0009 (3)	0.0056 (3)	0.0002 (3)
N1	0.029 (2)	0.0210 (15)	0.028 (2)	0.0045 (14)	0.0070 (16)	−0.0020 (14)
N2	0.0282 (19)	0.0216 (16)	0.034 (2)	−0.0003 (14)	0.0092 (17)	−0.0042 (14)
N3	0.031 (2)	0.0185 (15)	0.031 (2)	−0.0002 (14)	0.0089 (17)	0.0000 (14)
C1	0.025 (2)	0.0223 (19)	0.020 (2)	−0.0004 (16)	0.0026 (19)	0.0021 (16)
C2	0.037 (3)	0.0180 (19)	0.041 (3)	−0.0049 (17)	0.010 (2)	−0.0024 (18)
C3	0.033 (2)	0.0182 (18)	0.039 (3)	0.0005 (17)	0.010 (2)	−0.0044 (17)
C4	0.029 (2)	0.032 (2)	0.035 (3)	0.0010 (19)	0.009 (2)	−0.0055 (19)
C5	0.031 (3)	0.043 (3)	0.070 (4)	−0.001 (2)	0.016 (3)	−0.008 (3)
C6	0.024 (2)	0.0181 (19)	0.027 (2)	0.0029 (15)	0.0081 (19)	0.0022 (16)
P1	0.0330 (6)	0.0177 (5)	0.0230 (6)	−0.0002 (4)	0.0053 (5)	−0.0003 (4)
F1	0.0429 (17)	0.0558 (17)	0.0388 (18)	−0.0141 (13)	−0.0018 (14)	−0.0081 (13)
F2	0.0548 (18)	0.0329 (13)	0.0336 (16)	0.0120 (12)	0.0212 (14)	−0.0019 (11)
F3	0.084 (2)	0.0240 (12)	0.0376 (17)	0.0114 (13)	0.0255 (15)	0.0086 (11)
F4	0.0495 (17)	0.0613 (17)	0.050 (2)	−0.0274 (14)	0.0112 (15)	−0.0192 (14)
F5	0.0579 (17)	0.0311 (12)	0.0329 (16)	0.0117 (12)	0.0193 (13)	−0.0003 (11)
F6	0.0568 (18)	0.0257 (12)	0.0277 (15)	0.0051 (11)	0.0080 (14)	0.0052 (10)

### Geometric parameters (Å, °)

**Table:**

Cu1—N1	1.998 (3)	C2—H2	0.9500
Cu1—N1^i^	1.998 (3)	C3—H3	0.9500
Cu1—N3	2.074 (3)	C4—C5	1.328 (6)
Cu1—N3^i^	2.074 (3)	C4—H4	0.9500
N1—C1	1.352 (5)	C5—H5A	0.9500
N1—C3	1.421 (4)	C5—H5B	0.9500
N2—C1	1.392 (5)	C6—H6A	0.9900
N2—C2	1.436 (5)	C6—H6B	0.9900
N2—C4	1.456 (5)	P1—F6	1.625 (2)
N3—C6	1.516 (5)	P1—F1	1.631 (3)
N3—H3A	0.9200	P1—F4	1.634 (3)
N3—H3B	0.9200	P1—F2	1.638 (3)
C1—C6	1.531 (5)	P1—F3	1.638 (3)
C2—C3	1.375 (6)	P1—F5	1.658 (3)
			
N1—Cu1—N1^i^	180.00 (13)	C5—C4—N2	124.9 (4)
N1—Cu1—N3	82.53 (13)	C5—C4—H4	117.6
N1^i^—Cu1—N3	97.47 (13)	N2—C4—H4	117.6
N1—Cu1—N3^i^	97.47 (13)	C4—C5—H5A	120.0
N1^i^—Cu1—N3^i^	82.53 (13)	C4—C5—H5B	120.0
N3—Cu1—N3^i^	180.0	H5A—C5—H5B	120.0
C1—N1—C3	106.1 (3)	N3—C6—C1	106.0 (3)
C1—N1—Cu1	114.2 (2)	N3—C6—H6A	110.5
C3—N1—Cu1	139.7 (3)	C1—C6—H6A	110.5
C1—N2—C2	105.9 (3)	N3—C6—H6B	110.5
C1—N2—C4	127.2 (3)	C1—C6—H6B	110.5
C2—N2—C4	126.8 (3)	H6A—C6—H6B	108.7
C6—N3—Cu1	112.4 (2)	F6—P1—F1	89.70 (14)
C6—N3—H3A	109.1	F6—P1—F4	90.34 (15)
Cu1—N3—H3A	109.1	F1—P1—F4	179.76 (17)
C6—N3—H3B	109.1	F6—P1—F2	90.94 (13)
Cu1—N3—H3B	109.1	F1—P1—F2	89.76 (15)
H3A—N3—H3B	107.8	F4—P1—F2	90.48 (15)
N1—C1—N2	111.5 (3)	F6—P1—F3	179.61 (16)
N1—C1—C6	120.8 (3)	F1—P1—F3	90.11 (15)
N2—C1—C6	127.7 (3)	F4—P1—F3	89.84 (15)
C3—C2—N2	106.8 (3)	F2—P1—F3	89.41 (13)
C3—C2—H2	126.6	F6—P1—F5	89.31 (13)
N2—C2—H2	126.6	F1—P1—F5	90.23 (15)
C2—C3—N1	109.6 (4)	F4—P1—F5	89.53 (14)
C2—C3—H3	125.2	F2—P1—F5	179.75 (14)
N1—C3—H3	125.2	F3—P1—F5	90.34 (13)
			
N1^i^—Cu1—N1—C1	−64.0 (4)	C4—N2—C1—N1	−176.5 (4)
N3—Cu1—N1—C1	−6.8 (3)	C2—N2—C1—C6	−177.1 (4)
N3^i^—Cu1—N1—C1	173.2 (3)	C4—N2—C1—C6	5.6 (6)
N1^i^—Cu1—N1—C3	113.2 (6)	C1—N2—C2—C3	−0.4 (4)
N3—Cu1—N1—C3	170.4 (4)	C4—N2—C2—C3	176.9 (4)
N3^i^—Cu1—N1—C3	−9.6 (4)	N2—C2—C3—N1	−0.1 (5)
N1—Cu1—N3—C6	16.6 (3)	C1—N1—C3—C2	0.6 (5)
N1^i^—Cu1—N3—C6	−163.4 (3)	Cu1—N1—C3—C2	−176.8 (3)
N3^i^—Cu1—N3—C6	−41 (3)	C1—N2—C4—C5	−173.1 (4)
C3—N1—C1—N2	−0.9 (4)	C2—N2—C4—C5	10.1 (7)
Cu1—N1—C1—N2	177.2 (2)	Cu1—N3—C6—C1	−21.4 (4)
C3—N1—C1—C6	177.2 (3)	N1—C1—C6—N3	17.6 (5)
Cu1—N1—C1—C6	−4.7 (5)	N2—C1—C6—N3	−164.7 (4)
C2—N2—C1—N1	0.8 (4)		

Symmetry codes: (i) −*x*, −*y*, −*z*.
